# Supplementary material for: SyNDI: synchronous network data integration framework
Source: BMC Bioinformatics. 2018 Nov 6;19:403. doi: 10.1186/s12859-018-2426-5 (PMC6219086; doi:10.1186/s12859-018-2426-5)
Supplement: Supplementary file 5 — Files of the example presented in Additional file 4. This additional file is a zip package that contains all files used in Additional file 4. Detailed descriptions of each of these files are presented in a README file included in this zip package. (GZ 30411 kb) [file 12859_2018_2426_MOESM5_ESM.gz › SyNDITools/Meme2Fimo/meme_4.9.0_4.tar.gz!/meme_4.9.0/doc/dreme.html]

DREME - a motif discovery tool


# The MEME Suite

## Motif-based sequence analysis tools

### Usage:

`dreme [options] -p <sequence file> [-n <background sequence file>]`

### Description

DREME (Discriminative Regular Expression Motif Elicitation) finds
relatively short motifs (up to 8 bases) fast, and can perform
*discriminative* motif discovery if given a negative set, consisting
of sequences unlikely to contain a motif of interest that is however
likely to be found in the main ("positive") sequence set. If you do not
provide a negative set the program shuffles the positive set to provide a
background (in the role of the negative set).

The input to DREME is one or two sets of DNA sequences.
The program uses a Fisher Exact Test to determine significance of each
motif found in the postive set as compared with its representation in the
negative set, using a significance threshold that may be set on the
command line.

DREME achieves its high speed by restricting its search to regular
expressions based on the IUPAC alphabet representing bases and ambiguous
characters, and by using a heuristic estimate of generalised motifs'
statistical significance.

### Inputs

#### Sequence file (positives)

A collection of sequences in FASTA
format. The sequences should all be approximately the same length.

### Optional Inputs

#### Background Sequence File (negatives)

A collection of sequences in FASTA
format. The sequences should be approximately the same length as the
positive sequences.

If these sequences are not supplied then the program will shuffle
the positive sequences and use that set for the negative sequences.

### Output

DREME writes an XML file to the output folder and converts it into a
minimal MEME-formatted motif file and a human readable html file.

Additionally DREME can output motif logos if the -png and/or -eps options
are specified.

### Options:

| Option | Parameter | Description | Default Behaviour |
| --- | --- | --- | --- |
| Output | | | |
| --- | --- | --- | --- |
| -o | name | Create a folder called name and write output files in it. This option is not compatible with -oc as only one output folder is allowed. | The program behaves as if `-oc dreme_out` had been specified. |
| -oc | name | Create a folder called name but if it already exists allow overwriting the contents. This option is not compatible with -o as only one output folder is allowed. | The program behaves as if `-oc dreme_out` had been specified. |
| -png |  | Output motif logo images in portable network graphics (png) format. This format is useful for display on websites. | Images are not output in png format. |
| -eps |  | Output motif logo images in Encapsulated Postscript (eps) format. This format is useful for inclusion in publications as it is a vector graphics format and can be easily scaled. | Images are not output in eps format. |
| General | | | |
| --- | --- | --- | --- |
| -g | ngen | Set the number of REs to generalize. Increasing ngen will make the search more through at some cost in speed. | DREME will generalise 100 REs. |
| -s | seed | Set the seed for the random number generator used to shuffle the sequences. When the -n option is given the negative sequences will be used instead of shuffling. | The random number generator is initilised with a value of 1. |
| Stopping Conditions | | | |
| --- | --- | --- | --- |
| -e | e | Stop searching for motifs when the next motif's *E*-value is > e. | Stop discovering motifs if the *E*-value threshold of 0.05 is exceeded. |
| -m | m | Stop searching for motifs when m motifs have been found. | There is no limit on the number of discovered motifs. |
| -t | t | Stop searching for motifs when t seconds have elapsed. | There is no limit on the time taken to discover motifs. |
| Set Core Motif Width | | | |
| --- | --- | --- | --- |
| -mink | mink | Set the minimum width of the motif core. | A minimum core width of 3 is used. |
| -maxk | maxk | Set the maximum width of the motif core. | A maximum core width of 7 is used. |
| -k | k | Set the width of the motif core. This sets minimum width and maximum width to the same number. | The defaults for minimum and maximum width are used. |
| Miscellaneous | | | |
| --- | --- | --- | --- |
| -desc | description | Include the text description in the HTML output. | No description in the HTML output. |
| -dfile | desc file | Include the first 500 characters of text from the file desc file in the HTML output. | No description in the HTML output. |
| -verbosity | 1\|2\|3\|4\|5 | A number that regulates the verbosity level of the output information messages. If set to 1 (quiet) then it will only output error messages whereas the other extreme 5 (dump) outputs lots of mostly useless information. | The verbosity level is set to 2 (normal). |
| -h |  | Display a usage message and exit. | Run as normal |
| Experimental (use at own risk) | | | |
| --- | --- | --- | --- |
| -l |  | Print list of enrichment of all REs tested. |  |
